# Aggressive Mucormycosis with Extensive Craniofacial Involvement: A Case Report of Radical Surgical Management and Prosthetic Rehabilitation

**DOI:** 10.3390/reports8030187

**Published:** 2025-09-20

**Authors:** Alice Marzi Manfroni, Francesco Arcuri, Alessia Spinzia, Marjon Sako, Bernardo Bianchi, Francesco Laganà

**Affiliations:** 1 Maxillofacial Surgery Unit, IRCCS Ospedale Policlinico San Martino, 16132 Genoa, Italy; 2 Surgery Unit, University Trauma Hospital, 1016 Tirana, Albania

**Keywords:** mucormycosis, maxillectomy, reconstructive surgery, orbital exenteration, prosthesis and implants, case report

## Abstract

**Background and Clinical Significance:** Mucormycosis is a rare but potentially fatal opportunistic fungal infection with high morbidity and mortality rates despite aggressive treatment. Rhinocerebral mucormycosis represents the most common form, requiring prompt recognition and multidisciplinary management. **Case Presentation:** We report a 60-year-old female with glucose intolerance who developed extensive rhinocerebral mucormycosis involving the right maxillary sinus, orbit, and skull base. Despite initial antifungal therapy with amphotericin B, rapid disease progression necessitated radical surgical intervention including complete right hemimaxillectomy, orbital enucleation, and partial sphenoid bone resection with carotid siphon exposure. Initial reconstruction using a free scapular osteocutaneous flap failed due to vascular compromise, requiring salvage coverage with a temporalis muscle flap. Postoperatively, the patient recovered without cerebrovascular complications. Long-term rehabilitation involved implant-supported prosthetic reconstruction with osseointegrated implants placed in the remaining maxilla and fabrication of a custom obturator prosthesis to restore facial support and masticatory function. **Conclusions:** This case demonstrates the aggressive nature of mucormycosis requiring extensive surgical resection and highlights the challenges of reconstruction in infected tissues. While free flap reconstruction offers theoretical advantages, local tissue options provide reliable coverage when microvascular procedures fail. Comprehensive multidisciplinary care including prosthetic rehabilitation can achieve satisfactory functional outcomes following radical resection.

## 1. Introduction and Clinical Significance

Mucormycosis is a rare but potentially fatal opportunistic fungal infection caused by fungi belonging to the order Mucorales, with an estimated incidence of 1.7 cases per million population annually [1,2] The pathogen demonstrates remarkable angioinvasive properties, characterized by hyphal invasion of blood vessel walls, resulting in thrombosis, tissue necrosis, and rapid dissemination to adjacent anatomical structures [3,4,5]. Rhinocerebral mucormycosis represents the most common clinical manifestation, accounting for approximately 39% of all cases, and predominantly affects patients with predisposing conditions such as diabetes mellitus, hematologic malignancies, solid organ transplantation, or prolonged corticosteroid therapy [6,7].

The clinical presentation of rhinocerebral mucormycosis typically begins with nonspecific symptoms including facial pain, nasal congestion, and headache, which can rapidly progress to involve orbital and intracranial structures [8,9]. The maxillary sinus represents a common site of initial involvement, with potential extension to the ethmoid and sphenoid sinuses, orbit, and skull base [10,11]. Characteristic clinical findings include unilateral facial swelling, proptosis, ophthalmoplegia, and visual disturbances, which may progress to complete vision loss if left untreated [7]. The radiological spectrum demonstrates heterogeneous signal characteristics on magnetic resonance imaging, with distinctive features including tissue necrosis, bone destruction, and the pathognomonic “black turbinate sign” on computed tomography [9]. Diagnosis relies on a combination of clinical suspicion, characteristic imaging findings, and definitive histopathological confirmation demonstrating broad, ribbon-like, nonseptate hyphae with right-angle branching patterns [4,6]. However, diagnostic challenges frequently arise due to the nonspecific initial presentation and potential confusion with other sinonasal pathologies, often resulting in delayed recognition and treatment initiation [6]. While mucormycosis traditionally affects immunocompromised individuals, emerging reports describe cases in immunocompetent patients, particularly those with underlying diabetes mellitus or metabolic disorders [11,12].

Treatment of rhinocerebral mucormycosis requires a multidisciplinary approach combining aggressive antifungal therapy with radical surgical debridement [3,4]. First-line antifungal therapy typically consists of amphotericin B formulations, with newer agents such as posaconazole and isavuconazole serving as alternative or salvage therapies [1,4]. Surgical intervention remains crucial for disease control, ranging from endoscopic debridement in limited cases to extensive resection including orbital exenteration and skull base reconstruction in advanced disease [10,12]. Recent studies have demonstrated advantageous outcomes with the combination of topical amphotericin B and systemic antifungal therapy [13,14]. Despite optimal treatment, mortality rates remain substantial, ranging from 25–62% depending on the extent of disease and underlying patient factors [2]. The extensive nature of required surgical resection often necessitates complex reconstructive procedures, with recent advances in digital technology and prosthetic rehabilitation offering improved functional and aesthetic outcomes [12,15,16]. The reconstruction of extensive craniofacial defects following radical mucormycosis resection presents challenges extending beyond clearance: inadequate reconstruction can result in life-threatening complications including cerebrospinal fluid leakage, meningitis, and exposure of vital structures. Moreover, the functional and psychosocial implications profoundly impact quality of life, affecting mastication, deglutition, speech, and facial expression while causing aesthetic disfigurement leading to social isolation and psychological distress. The craniofacial region’s central role in human interaction necessitates sophisticated reconstructive approaches utilizing complex free tissue transfers or carefully planned local and regional flaps when microvascular reconstruction is contraindicated. These elaborate strategies, often combined with implant-supported prosthetic rehabilitation, aim to restore structural integrity, prevent complications, and rehabilitate critical functional and aesthetic parameters essential for patient reintegration [16].

## 2. Case Presentation

A 60-year-old Caucasian female with a medical history significant for glucose intolerance managed with oral hypoglycemic agents presented to the emergency department of IRCCS Ospedale Policlinico San Martino, Genoa, Italy with a three-day history of progressively worsening right-sided facial pain, cephalgia, and diplopia. The patient reported no recent trauma, dental procedures, or upper respiratory tract infections. Her glycemic control had been stable, and she denied any other immunocompromising conditions.

Physical examination revealed marked swelling and erythema of the right hemifacial region, particularly involving the infraorbital and temporal areas. Notable proptosis of the right eye was observed with limited extraocular movements and decreased visual acuity. Intraoral examination demonstrated erythematous and edematous tissues overlying the right maxillary region with no obvious intraoral lesions. Cranial nerve examination was otherwise unremarkable at presentation. The patient underwent comprehensive preoperative ophthalmological evaluation including visual acuity testing, ocular motility examination (OME), and Hess-Lancaster testing, which confirmed reduced visual acuity and impaired ocular motility.

**Figure 1:**
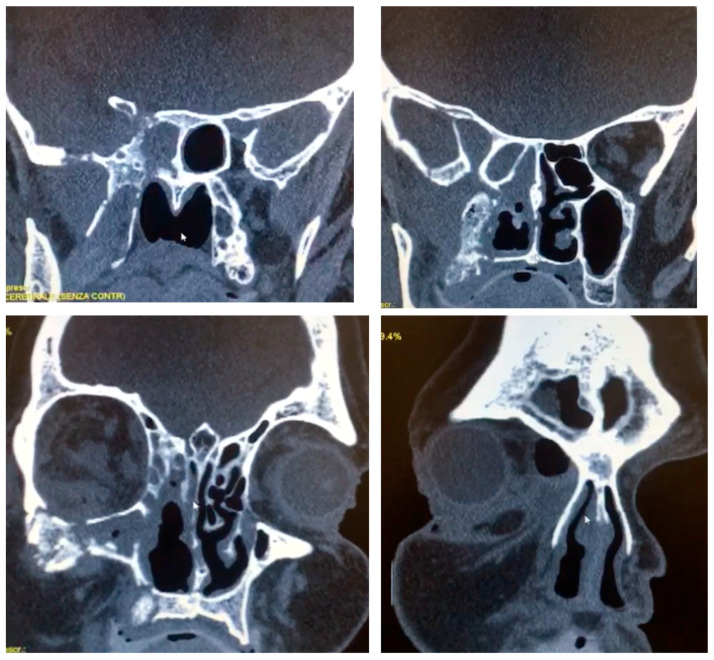
CT scan with contrast enhancement showing osteolytic changes involving the right maxillary walls, orbital floor, and medial orbital wall, reaching the sphenoid bone and skull base.

Computed tomography (64-slice MDCT scanner, Siemens Healthineers^®^, Berlin, Germany) with contrast enhancement, 1.25 mm slice thickness and 262,144 voxels per slice revealed extensive opacification of the right maxillary sinus with associated osteolytic changes involving the maxillary walls, orbital floor, and medial orbital wall (Figure 1). Magnetic resonance imaging (1.5 Tesla MRI, Siemens Healthineers^®^, Berlin, Germany) demonstrated heterogeneous signal intensity within the affected sinuses with evidence of orbital fat infiltration and extension into the sphenoid sinus with skull base involvement. The imaging findings were highly suggestive of an aggressive infectious process with characteristics consistent with mucormycosis.

Tissue biopsy obtained from the right maxillary sinus via endoscopic approach revealed broad, nonseptate hyphae with right-angle branching patterns characteristic of Mucorales species. Histopathological examination demonstrated extensive tissue necrosis with angioinvasion. Periodic acid–Schiff and Grocott–Gomori methenamine-silver staining confirmed the presence of fungal elements consistent with mucormycosis. Immediate antifungal therapy was initiated with intravenous amphotericin B (1.5 mg/kg/day). Despite aggressive medical treatment, clinical deterioration continued over the subsequent 48 h with progression of orbital involvement and development of complete vision loss in the affected eye.

**Figure 2:**
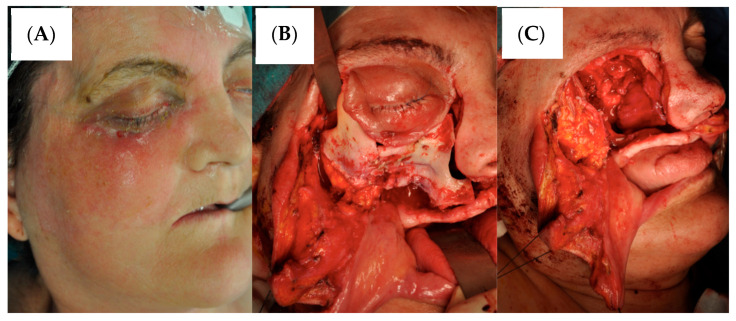
(**A**) Preoperative clinical photograph showing facial edema and erythema in the right malar and periorbital regions. (**B**) Intraoperative photograph illustrating surgical exposure via Weber-Ferguson incision approach, demonstrating visualization of the right hemimaxilla and its osteotomy. (**C**) Intraoperative photograph showing the surgical defect following right hemimaxillectomy with orbital exenteration, demonstrating the extent of bone and soft tissue resection.

Given the rapid disease progression and failure of medical management, emergency surgical intervention was undertaken. A comprehensive radical debridement was performed under general anesthesia, including complete right hemimaxillectomy with removal of all affected maxillary bone and soft tissues, right orbital exenteration with careful attention to hemostasis of the central retinal artery to prevent intracranial hemorrhage, partial sphenoid bone resection with controlled exposure of the carotid siphon and extensive debridement of all necrotic and infected tissues (Figure 2). The surgical cavity was copiously irrigated with amphotericin B solution, and meticulous hemostasis was achieved throughout the procedure.

Initial reconstruction was attempted using a free scapular osteocutaneous flap with microvascular anastomosis to recipient vessels in the neck. However, vascular compromise developed at the anastomosis site within 24 h postoperatively, resulting in complete flap failure and necessitating immediate return to the operating room for removal of the necrotic tissue and concurrent temporalis muscle flap rotation to provide coverage of the extensive surgical defect and protection of the exposed carotid artery. A secondary reconstruction with a new microvascular free flap was planned once the patient’s clinical condition stabilized. However, considering the patient’s significant physical and psychological burden from the underlying disease, prolonged hospitalization, and intensive treatments, she declined to proceed with the third surgical intervention.

No evidence of cerebrovascular complications was recorded despite the carotid exposure. Intravenous amphotericin B therapy was continued for six weeks, followed by oral posaconazole maintenance therapy. Serial imaging studies demonstrated no evidence of residual or recurrent infection in the six months after surgery.

**Figure 3:**
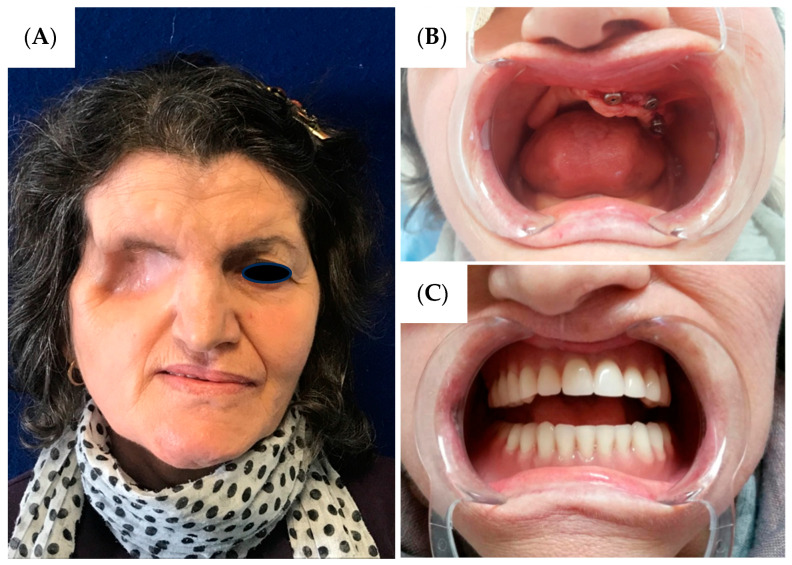
(**A**) Six-months post-operative photographs of the patient and dental prosthetic rehabilitation (**C**) after left maxillary implant positioning (**B**).

Comprehensive rehabilitation was initiated three months postoperatively. The patient was completely edentulous preoperatively, and implant-prosthetic treatment was commenced following complete healing. Due to the extensive maxillary defect, functional and aesthetic restoration required a multidisciplinary approach. Three osseointegrated dental implants measuring 4.5 × 12 mm were placed in the remaining left maxilla (Figure 3B), and a custom maxillary obturator prosthesis was fabricated extending across the surgical defect to restore facial support, speech articulation, and masticatory function (Figure 3).

The patient demonstrated overall satisfaction with her morphological restoration outcome. During the initial six-month postoperative period, significant psychological adaptation challenges were encountered regarding the facial aesthetic alterations, particularly the ocular loss. However, with progressive tissue healing and implementation of the comprehensive morpho-functional rehabilitation protocol, gradual psychological acceptance was achieved, culminating in patient satisfaction with the result. Functional recovery proved excellent, with restoration of masticatory function, speech articulation, and complete resumption of social activities, effectively reestablishing an acceptable quality of life despite the extensive surgical resection.

## 3. Discussion

This case exemplifies the profound challenges inherent in managing advanced rhinocerebral mucormycosis, a condition that demands aggressive intervention despite substantial associated risks. The rapid progression of disease despite seemingly adequate antifungal therapy underscores the relentless nature of this invasive infection and the critical importance of early surgical intervention when medical management proves insufficient [4,6]. The extensive involvement of maxillary, orbital, and skull base structures in our patient necessitated radical resection that inevitably resulted in significant functional and aesthetic morbidity, highlighting the devastating impact of advanced mucormycosis on patient quality of life.

The management approach aligns with recent literature emphasizing the necessity of aggressive surgical debridement in rhino-orbital mucormycosis [7]. The decision for early orbital exenteration given preoperative visual acuity reduction and motility deficits demonstrated on Hess–Lancaster testing is consistent with established protocols advocating prompt orbital intervention when visual compromise is evident. Other reported cases have demonstrated successful rehabilitation following extensive orbital and maxillary defects using digital technology for prosthetic reconstruction [9], supporting the multidisciplinary approach combining implant-supported prosthetics with obturator therapy. Furthermore, intraoperative topical amphotericin B application to the surgical bed was incorporated, as recently described in the literature for enhanced local antimicrobial control [13,14]. The rapid progression requiring urgent intervention contrasts with some literature reports of indolent presentation [10], emphasizing the variable clinical behavior of mucormycosis and the need for individualized treatment strategies. The decision to proceed with such extensive surgical intervention, including orbital enucleation and skull base resection with carotid siphon exposure, represents a complex risk-benefit analysis balancing life-threatening infection against equally serious surgical complications. The proximity of vital neurovascular structures at the skull base creates inherent risks of cerebrovascular injury, cranial nerve damage, and cerebrospinal fluid leakage during resection [5,7]. In the presented case, the controlled exposure of the carotid artery during sphenoid bone resection required meticulous surgical technique and continuous monitoring to prevent potentially catastrophic vascular complications. The successful management of this exposure without neurological sequelae demonstrates the feasibility of such aggressive approaches when performed by experienced multidisciplinary teams.

The profound aesthetic and functional deficits resulting from hemimaxillectomy and orbital enucleation extend far beyond mere anatomical loss [17,18]. The disruption of essential functions including mastication, deglutition, speech articulation, and facial expression creates substantial challenges for patient rehabilitation and social reintegration. The psychological impact of facial disfigurement, particularly involving orbital loss, cannot be understated and requires comprehensive psychosocial support throughout the treatment continuum. These considerations emphasize why reconstruction becomes not merely an aesthetic concern but a fundamental component of comprehensive patient care. The failure of free flap reconstruction highlights specific challenges in the setting of mucormycosis-compromised tissues. The angioinvasive nature of the pathogen and associated vascular damage likely contributed to anastomotic failure, necessitating temporalis muscle flap coverage for reliable protection of vital structures including the exposed carotid artery. While microvascular free tissue transfer would have provided superior aesthetic and functional outcomes [15,18], the patient declined additional reconstruction following the initial flap failure due to physical and emotional exhaustion from the prolonged treatment course. Long-term functional rehabilitation through implant-supported prosthetic reconstruction represents a critical component of comprehensive care following extensive maxillary resection. The integration of osseointegrated implants with custom prosthetic devices allows for meaningful restoration of facial support, masticatory function, and speech clarity. This multidisciplinary approach, combining surgical expertise with advanced prosthodontic techniques, demonstrates the potential for achieving satisfactory outcomes even following the most extensive resections.

The patient’s relatively good health status prior to infection onset emphasizes an important clinical lesson: mucormycosis can affect individuals with seemingly minor predisposing factors. Even well-controlled glucose intolerance may provide sufficient immunocompromise to allow fungal proliferation, particularly in the presence of additional stressors. This case underscores the importance of maintaining high clinical suspicion for opportunistic infections in any patient with metabolic disorders, regardless of their apparent degree of control.

From a broader perspective, this case illustrates the evolution of mucormycosis management from a uniformly fatal condition to one where aggressive multimodal therapy can achieve meaningful survival and functional outcomes. However, the extensive morbidity associated with successful treatment raises important questions about quality of life considerations and the need for comprehensive rehabilitation services. The psychological, social, and economic impacts of such extensive treatment require careful consideration and planning from the outset of care.

## 4. Conclusions

Rhinocerebral mucormycosis remains a challenging clinical entity requiring prompt recognition and aggressive multidisciplinary management. This case demonstrates that even patients with minor predisposing factors can develop life-threatening infections requiring extensive surgical intervention. Early diagnosis, aggressive antifungal therapy, and radical surgical debridement remain the cornerstones of successful treatment. When extensive reconstruction is required, local tissue transfer options may provide more reliable outcomes than free tissue transfer in the setting of active infection. Comprehensive rehabilitation through prosthetic reconstruction can achieve satisfactory functional and aesthetic outcomes following radical resection.

## Data Availability

The original contributions presented in this study are included in the article. Further inquiries can be directed to the corresponding author.
